# Open-WiSe: A Solar Powered Wireless Sensor Network Platform

**DOI:** 10.3390/s120608204

**Published:** 2012-06-13

**Authors:** Apolinar González, Raúl Aquino, Walter Mata, Alberto Ochoa, Pedro Saldaña, Arthur Edwards

**Affiliations:** Faculty of Mechanical and Electrical Engineering, University of Colima, Av., Universidad # 333, 28000 Colima, Mexico; E-Mails: aquinor@ucol.mx (R.A.); wmata@ucol.mx (W.M.); aochoa@ucol.mx (A.O.); pedro_saldana@ucol.mx (P.S.); arted@ucol.mx (A.E.)

**Keywords:** solar-powered wireless sensor platform, real-time kernel, sleep and wakeup strategies, 802.15.4

## Abstract

Because battery-powered nodes are required in wireless sensor networks and energy consumption represents an important design consideration, alternate energy sources are needed to provide more effective and optimal function. The main goal of this work is to present an energy harvesting wireless sensor network platform, the Open Wireless Sensor node (WiSe). The design and implementation of the solar powered wireless platform is described including the hardware architecture, firmware, and a POSIX Real-Time Kernel. A sleep and wake up strategy was implemented to prolong the lifetime of the wireless sensor network. This platform was developed as a tool for researchers investigating Wireless sensor network or system integrators.

## 1. Introduction

Today, wireless sensor nodes are key components of wireless sensor networks (WSNs), which, in themselves, have become an important field of study. WSNs have evolved significantly over the past decade in the areas of computing capacity, sensor resources, communication, energy storage, and cost.

Because WSNs require battery-powered nodes and energy consumption is a primary factor in the design of WSNs, alternate energy sources are needed to permit them to function more effectively and for longer periods of time. Solar energy has become more attractive recently because of its environmental benefits and because the efficiency of photovoltaic cells has increased significantly in the past few years. Solar energy is derived from Nature's greatest renewable resource and it is non-toxic in nature. Solar cells can be utilized to power the sensor as well as to charge the batteries for WSNs [1], where their design goal is to provide autonomy lifetime (for wireless sensor nodes), making WSNs more valuable in terms of versatility and longevity.

Today's sensor platforms can be classified into two major categories [2]. The first category is related to an application-specific platform which usually consists of a low power processor module, a communication module, and a sensor module. This type of platform is highly efficient in small spaces. However, the time required developing an application as well as the addition of new features and subsequent cost are important considerations that need be taken into account. The second category is a general platform which is designed for WSN prototyping, but it has the disadvantage of consuming significantly more power and having a considerably larger physical size [3]. This work presents a new solar-powered wireless sensor network platform for the feasible development of WSN applications consisting of an open hardware platform and A POSIX Real-Time Kernel, an open source real-time kernel. To test the concept, an application was developed where sensor nodes employed a sleep and wake up strategy were needed to prolong the lifetime of the WSN. The contributions of this paper are two-fold: (1) it provides the design of an open hardware solar-powered WSN platform, and (2) it adds new user libraries to The POSIX Real-Time Kernel for WSN applications.

This paper is organized as follows: Section 2 presents related work. Section 3 then goes on to presents a model for our solar power system. Section 4 follows with a discussing of sleep and wake up policies to prolong the life of WSNs by using solar power to complement or replace conventional battery power sources. Section 5 introduces the reader to The POSIX Real-Time Kernel and shows readers how to use the libraries that we have developed, our prototype, and results are given in Section 6, where we transition to Section 7 and present our conclusions and future work.

## 2. Related Work

Research groups have developed various different platforms, some of which have very specific characteristics depending on application requirements. In [4] propose a hybrid wireless sensor networks architecture based on the Tmote sky and Fox board platform. Whereas in [5], probably the most well-known platform is the Mica series from UC Berkeley. In [3], the SENTIO platform is presented as a hardware platform for rapid prototyping of WSN. Both Mica and SENTIO have similar computational power and consume approximately the same power. However, unlike these, Open-WiSe has greater computational power and similar power consumption because it is designed for either novices or expert WSN designers.

Open-WiSe consumes approximately the same amount of power as Mica and SENTIO. It does so by disconnecting the Xbee wireless module and solely operating with the CC2420 radio. Furthermore, it also contains a solar charger module in the same PCB. Other recent platforms include Telos [6] from UC Berkeley and XYZ [7] from Yale University, which consume significantly less power than many conventional platforms.

While Open-WiSe has an internal real time clock that is embedded in the microcontroller, the XYZ platform has a supervisor circuit outside the processor that is used to shut down the peripherals on the board. Related to solar powered designs, the Trio [8] and Heliomote platforms [9], two leading designs, have different architectural designs which provide them with important advantages. A complete analysis between Trio and Heliomote can be found in [10]. However, in brief, whereas Trio has a two-level energy storage system (1.8 V and 2.75 V from initial and final level of energy) and employs software-controlled battery charging, Heliomote and Open-WiSe employ single-level energy storage and hardware-controlled battery charging. Importantly, regardless of their differing features, all of these platforms suffer from performance limitations when working in conjunction with solar powered systems; Trio platform work only a few hours with a 100% duty-cycle if the sun-light is present and the rest of time is 20% to 40% of duty-cycle which is low.

In order for a wireless sensor network to work independently for an infinite time, devices or systems have been developed that allow you to collect solar energy and convert it into a variable voltage output to driving multiple loads. In [11] an energy harvesting chip to scavenge energy from artificial light to charge a wireless sensor node was developed. This chip core uses a miniature transformer with a nano-ferrofluid magnetic to convert the harvested energy loaded. The authors mentioned that in their measurements that proposed system achieved good results, even when one has a low luminosity of 240 LUX, and 54% of the energy collected can be saved by the feedback control of the sensor network described. The amount of power harvesting in the experimental setup is 15 mA and only 5 mA is consumed in state on, but the reference does not mention exactly how much energy is consumed in the off state.

In other work similar to the last design, a scheme that collects energy using solar panels but employing 34 W fluorescent lamps as an energy source, which are commonly found in the halls or rooms of hospitals was developed [12]. The energy harvesting module consists of a power management circuit that is connected to a group of ultracapacitors that store energy and manage the power supply to the nodes. After analyzing this work, we see that the total amount of energy used in the off and on states is 21 mA and the off state consumes only 9 mA, which is similar to the power in the on state of 12 mA. From these data can be summarized that energy employed in off state is wasted.

A solar cell-based power supply for an agricultural environment monitoring server system for monitoring information concerning an outdoors agricultural production environment utilizing Wireless Sensor Network (WSN) technology is implemented in [13]. This technology could contribute to increasing crop yields and improving quality in the agricultural field by supporting the decision making of crop producers through analysis of the collected information.

## 3. System Architecture

**Figure 1.**
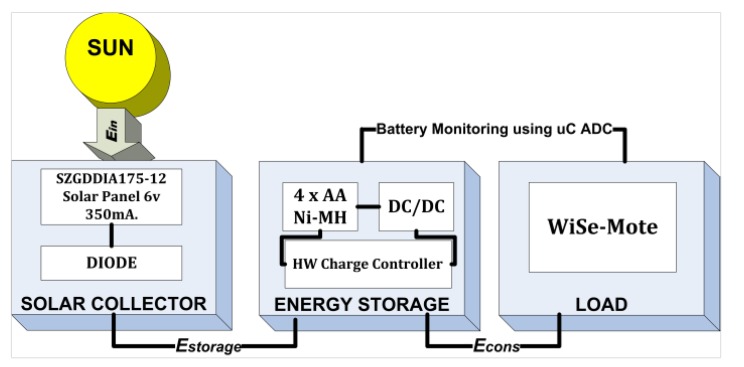
Model for a solar powered system.

Figure 1 shows our solar powered system which is divided into three sections: (1) the solar collector, (2) the energy storage, and (3) the load. The operation of each component is described separately below.

### 3.1. Solar Collector

Solar energy is collected by the solar collector module. During darkness, the solar cell is not an active device; instead, it works as a diode and produces neither a current nor a voltage. For our work, we consider a model of moderate complexity with regards to the photovoltaic solar cell [14]. The amount of current generated (*I_c_*) depends on the intensity of solar radiation (*Gr*, in watt/m^2^) and is expressed in Equation (1):

(1) $$I_{c}(Gr)=I_{L}(Gr)-I_{0}(.)-\frac{\left(V_{c}-I_{c}R_{s}\right)}{R_{\text{sh}}}$$

where *I*_0_(.) is a function of diode saturated current, *R_s_* is a series resistance which gives a more accurate shape between the maximum power point and the open circuit voltage. This represents the internal loss due to current flow (resistance). *R_sh_* is a shunt resistance and is in parallel with the diode. This helps account to leakage to the ground, a factor commonly neglected. Load Current (*I_L_*) can be obtained from Equation (2):

(2) $$I_{L}(Gr)=(D_{0}+D_{1}T_{c})Gr$$

where *D*_0_ and *D*_1_ are constant coefficients, *T_c_* is the temperature of the solar cell and *Gr* is the intensity of solar radiation in watts/m^2^.

### 3.2. Energy Storage

The energy storage module is used to store energy from the solar collector in the batteries. It is also responsible for providing energy to the WiSe mote as well as controlling the battery charging circuit. We assume the total capacity of the battery as C and the battery state in a time slot as *c* ∈ {0,1,…,C}. A linear battery model [15] was considered to obtain the remaining battery capacity expressed in Equation (3):

(3) $$c=c^{\prime }-\int_{t_{0}}^{t_{0}+t}I\left(t_{0}\right)\text{dt}t$$

If *c*′ is the previous capacity and *I*(*t*_0_) is the instantaneous current consumed by the circuit at time *t*_0_, we can then estimate the current consumption [10] Inode of the WiSe-mote using Equation (4):

(4) $$I_{\text{node}}=R\cdot I_{\text{awake}}+\left(1-R\right)\cdot I_{\text{sleep}}$$

Thus, current consumption is expressed as a linear function of different duty-cycle rates *R* (0 < *R* < 1). Duty-cycling allows a sensor node to communicate with less energy consumption although, at times, with increased latency.

### 3.3. WiSe-Mote

**Figure 2.**
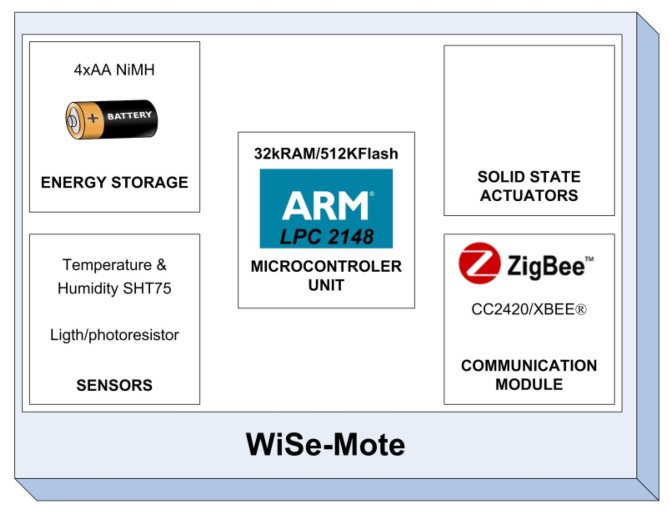
Model of the Open-WiSe mote.

Figure 2 shows the different modules that comprise the Wise-mote, whose system design faces three significant challenges, which include:

- Building a sufficiently lightweight, efficient hardware platform capable of monitoring and controlling physical variables.
- Incorporating software algorithms to achieve autonomous transfer sensing.
- Integrating subsystems such as microprocessor, sensor-actuator modules, and wireless networking into a fully functional wireless platform solution.

**Figure 3.**
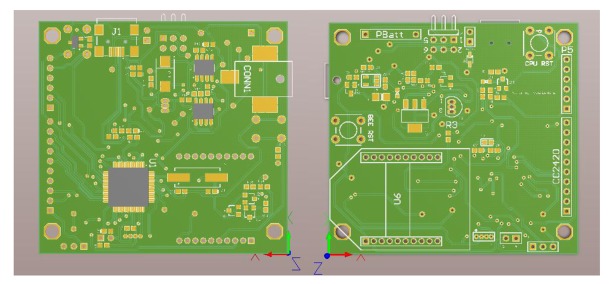
WiSe Mote PCB Design.

The system's master module is the processor, which is built around a 32-bit ARM7TDMI-S CPU with real-time emulation and embedded trace support. The actual microcontroller used is the LPC2148 which with its 512 K of high-speed flash memory and 32 K of SRAM data memory is capable of running a POSIX real-time kernel and many kinds of different routing algorithms. It also contains many interesting features including: a low-power Real-Time Clock (RTC) with independent power and a 32 kHz clock input; a 60 MHz maximum CPU clock available from programmable on-chip PLL; multiple serial interfaces including two UARTs; two fast I2C-bus (400 kbit/s); Serial Peripheral Interface (SPI) and Synchronous Serial Port (SSP) with buffering and variable data length capabilities; on-chip integrated oscillator operates with an external crystal from 1 MHz to 25 MHz and power saving modes that include Idle and Power-down. Therefore a USB 2.0 full-speed compliant device controller with 2 kB of endpoint RAM is embedded in the MCU. Figure 3 shows the WiSe mote PCB design which is 56 × 58 mm in size and of a two- layer type; the microcontroller package used is the 10 × 10 mm LQFP64. By using this kind of package it is possible to solder all components on the board manually, thereby saving time and cost for smaller volumes.

The communication module is based on the IEEE 802.15.4 compliant RF transceiver and the WiSe communication module contains two different radios. The first one is the CC2420 [16]; the communication between the MCU and the CC2420 is done via SPI interface. This radio only provides support for the Medium Access Control (MAC) layer and the Physical layer (PHY); consequently, the programmer is responsible for developing the routing algorithm (Network layer) and the application layer. The second one is the XBEE radio [17] by Digi International Inc. This radio is designed to operate under the ZigBee protocol and was added to the WiSe mote with the goal of developing a rapid WSN application. On the other hand, the CC2420 was added for programmers with a little more experience in WSN. Because of the energy consumption of the radios, just one of them can be used at a time.

The device is designed to operate with different types of power supplies. For the purpose of testing and development, it can be powered via a 5 V DC power supply or a USB data cable. However, for a real application it should be powered via batteries and solar cells. The sensor module is based on the SHT75 which integrates sensor elements plus signal processing capacities in a compact format that provides fully calibrated digital output. A unique capacitive sensor element is also used to measure relative humidity while the temperature is measured by a band-gap sensor. In addition, this module contains a Photoconductive Cell which is connected to the Analogue-Digital Converter (ADC). External Actuators can be connected to the platform via expansion headers.

## 4. Sleep-Wake up Strategies

Because a credible deployment of WSN is based on the energy management of sensor nodes and energy is a finite resource, different sleep and wake up polices can be used to prolong the WSN lifetime. We consider two variables (battery *l* and solar radiation *r*) in the sleep and wake up models. When the battery state is *l* and the solar radiation is *r*, the node can switch from the active to the sleep mode and vice versa. We use a random variable *N* to represent the mode of operation of the sensor (*i.e.*, *N* = sleep, *N* = active) hence from [12] the probability *P_a→_*_s_(*l*, *r*) = *Prob*(*N*(*t*) = sleep | *N*(*t* − 1) = active), from the sleep mode to the active mode *P_s→_*_a_(*l*, *r*) = *Prob*(*N*(*t*) = active | *N*(*t* − 1) = sleep). To test our concept, we selected a hybrid model which is composed of a combination of the battery and solar radiation models. We will present this information later in this paper.

### 4.1. Battery-State Strategy

If the battery capacity is below the threshold level *C_low_*, the node will change the state to sleep and will go back to the active state when the battery state reaches the threshold high level *C_hi_* which is expressed in Equation (5):

(5) $$\begin{array}{c} P_{a\to s}\left(l,r\right)=\left\{ \begin{array}{l} 1,c/C\leq c_{\text{low}}\\ 0,c/C>c_{\text{low}} \end{array}\right.\\ P_{s\to a}\left(l,r\right)=\left\{ \begin{array}{l} 1,c/C\geq c_{\text{hi}}\\ 0,c/C<c_{\text{hi}} \end{array}\right. \end{array},c_{\text{low}}>0$$

### 4.2. Solar Radiation Strategy

The amount of solar radiation depends on the environmental conditions and it is the main factor that determines the availability of radiant energy for the solar collector. If solar radiation is low, the photovoltaic cells of the panel cannot produce enough energy to charge the battery of the sensor node. Therefore, the nodes go into sleep mode or go into active as the energy produced increases in function of the solar radiation *r* given by Equation (6):

(6) $$\begin{array}{c} P_{a\to s}\left(l,r\right)=\left\{ \begin{array}{l} 1,r=0\\ 0,r>0 \end{array}\right.\\ P_{s\to a}\left(l,r\right)=\left\{ \begin{array}{l} 0,r=0\\ 1,r>0 \end{array}\right. \end{array}$$

Therefore, the probability of increasing battery capacity depends on the current from the solar cell and can be obtained from *P_charge_* = *I_c_*(*Gr*)/*I_node_*.

### 4.3. Hybrid Strategy

In this strategy, the solar radiation and the battery-state models were combined to obtain a hybrid model (7). For example, the sensor switches to sleep if the battery capacity is below the threshold level *C_low_* AND if the amount of solar radiation is null.

(7) $$\begin{array}{c} P_{a\to s}\left(l,r\right)=\{\begin{array}{c} 1,c/C\leq c_{\text{low}}\;\text{AND}\;r=0\\ 0,c/C>c_{\text{low}}\;\text{OR}\;r>0 \end{array}\\ P_{s\to a}\left(l,r\right)=\{\begin{array}{c} 1,c/C\geq c_{\text{hi}}\;\text{OR}\;r>0\\ 0,c/C<c_{\text{hi}}\;\text{AND}\;r=0 \end{array} \end{array},c_{\text{low}}>0$$

## 5. Software Design

### 5.1. A POSIX Real-Time Kernel

**Figure 4.**
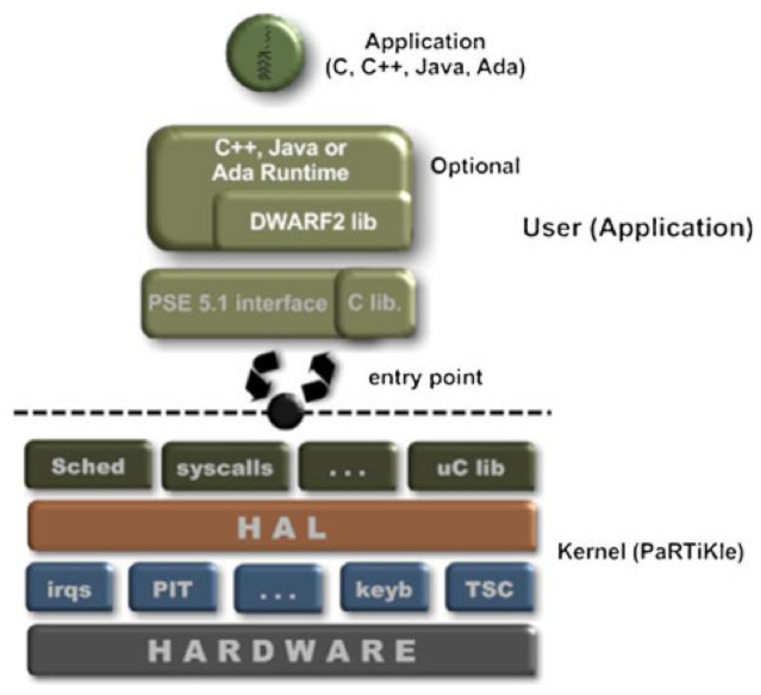
PaRTiKle OS Architecture [20].

The Figure 4 shows the architecture of the POSIX Real-Time Kernel that we called PaRTiKle [18–20], it has been designed to be compatible with POSIX and to support application development with real-time features like full preemptability, minimal interrupt latencies, synchronization primitives, scheduling policies, and interrupt handling mechanisms. PaRTiKle has a small footprint including a lightweight and energy-efficient scheduler, a user-level network stack and other components such as device drivers. One of its strengths is that developers can write the application code in standard ANSI C and compile it with GCC, avoiding the need to learn a specialized language or compiler. PaRTiKle's structure also provides several advantages over existing sensor network platforms, because it supports multiple execution environments, programming languages (Ada, C, C++, and Java), portability, configurability and maintenance.

Contrary to other small embedded RTOS, which are implemented as a library that is linked to the application, PaRTiKle has been designed as a real-time kernel with a clean and well-defined separation between the kernel and application execution spaces. All kernel services are provided via a single entry point, which improves the robustness and simplifies the portability PaRTiKle to other architectures and environments.

### 5.2. Building Process of Sensor Applications

**Figure 5.**
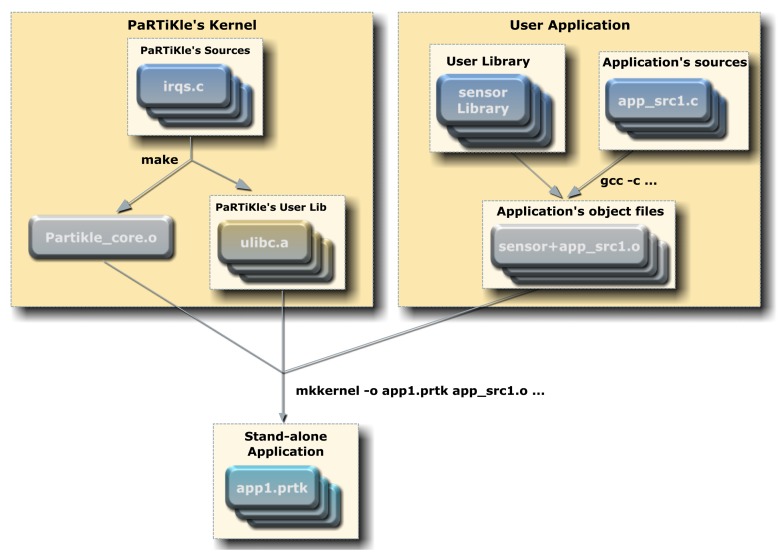
Building Process of Sensor Application.

Programmers can automatically generate a template for the end of the application by means of the autogen tool, which was designed to create program files that contain repetitive text with varied substitutions. The generated code makes use of a definition file which is completely separate from the template file; the use of this definition file increases the flexibility of the template implementation and the respective generated code. The platform has three main elements: (1) the development model, (2) the sensor library, and (3) the user application and PaRTiKle's Kernel. Figure 5 illustrates the development process of a sensor application running on PaRTiKle which makes use of a bash script named mkkernel. As part of the building process, the following steps must be performed: (1) the application previously generated with autogen and modified by the developer with the changes needed to end the user application must be linked with the sensor library, and (2) the resulting object is linked with the kernel object to create an executable file (*.prtk) containing all the components.

The mkkernel script requires the following parameters:

$ mkkernel -f <output><file1.o> [<file2.o> …]

where <output> is the name of the target executable once the process of building the application has concluded, and <file1.o>, <file2.o>, *etc.*, are the object files obtained when the application is compiled using GCC with the option -c. The steps performed by this script:

1. link the application against the user C library and the suitable run-time (the run-time is selected depending on the language used to implement the application).
2. turn every applications symbol into a local symbol, except user entry point.
3. link the script and the resulting object file together with the kernel object file to create the executable (*.prtk).

For example, consider the source file example.c; this file is compiled using GCC with the -c flag:

$ gcc -c example.c -I <headers>

where <headers> is the path to the PaRTiKle C user header files (ulibc/include).

The result of this compilation is a file called example.o. After that, we invoke the mkkernel script as follows:

$ mkkernel -o example example.o

The result is a binary file named example.prtk which is ready to be executed in the selected execution environment.

### 5.3. User Libraries for Sensor Applications

In this subsection we describe the way to employ PaRTiKle's user libraries to obtain the physical variables. Table 1 shows the primary functions that we developed. These functions are inside the user's library files and are used to obtain the actual values for the physical variables (temperature, relative humidity, and light). The code shown below is an example of how to use the sensor library. First we have to add the header file sensores.h. After this, we declare the variables where the values sent by the sensors will be stored. Notice that if a person wants to obtain the actual value of light he/she must first call the adcInit0_6() function.

#include <sensores.h>#include <LPC214x.h>#include <stdio.h>int main (int argc, char **argv) {  char buffer[100];  unsigned int x;  float temperature,humidity; adcInit0_6();  for(;;){   sht75_read(&temperature, &humidity);   x = adcRead0_6();  }  return 0;}

## 6. Implementation

To test the concept we developed a simple application where two wireless sensor nodes were used to measure the humidity, temperature, and light of a soccer field.

### 6.1. Measurement Setup

We used the Fluke 87-V multimeter to measure the consumption of the WiSe mote at different duty-cycle rates for one hour (e.g., radio duty-cycle 2%, radio awake time: 1,028 ms, message transmission interval: 5 s).

To measure the received signal strength indication (RSSI), one node sensor was declared the transmitter, sending 10 packages per second and measurements were taken every 5 m. The values of the physical variables were programmed to update every 250 ms. The second node acted as a receiver and forwarded the RSSI value to a personal computer. For this test no particular routing algorithm was programmed into the node. Only the PaRTiKle Operating System and a few lines of code were needed.

The maximum energy harvesting by solar cell in the tests was 2.09 W and its efficiency of conversion was 15% (e.g., Vmp: 5.8 V, Imp: 342 mA, Pmax: 2.09 W).

### 6.2. Results

As we can see in Tables 2 and 3 the difference between average current (*I_avg_*) and the estimated current (*I_node_*) is very small, thus the estimation of current consumption can be done using Equation (4). Also, we can see that the WiSe mote current consumption using the CC2420 radio is smaller than using the Xbee radio and that is because the Xbee radio contains an 8-bit microcontroller (9SGT60) with both the network and the application layer. In the case of XBEE radio transceiver, the system consumes an energy of 6.30 mA in sleep state and 69 mA in wake-up state, relationship that goes beyond the results presented in the cases analyzed in Section 2.

**Figure 6.**
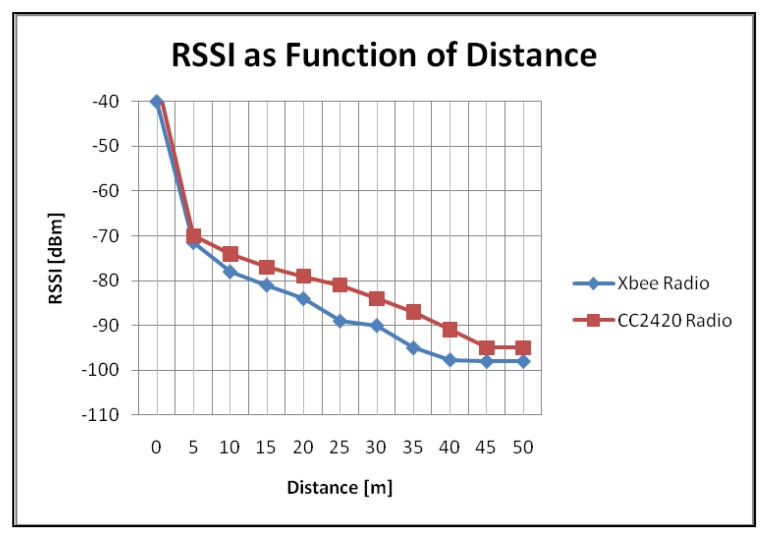
RSSI as function of distance.

**Figure 7.**
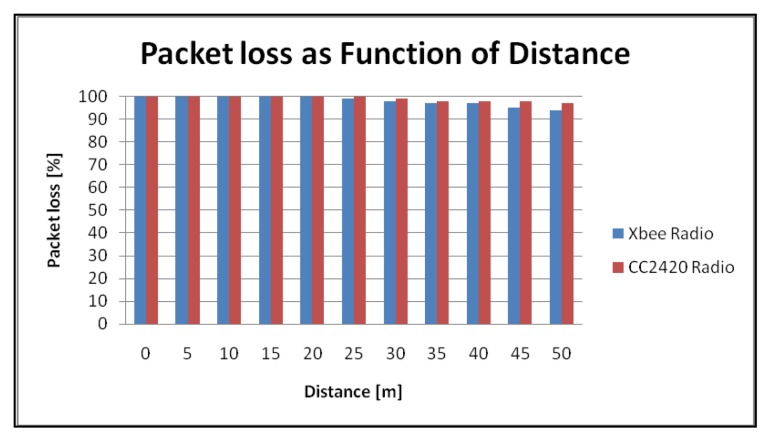
Packet loss as a function of distance.

Figure 6 shows the received signal strength as a function of the distance for the Xbee and the CC2420 radios. The measures were taken in an open environment with line of sight (soccer field) and a maximum transmit power output configuration in both radios. The graph in Figure 7 illustrates the packet loss during the measurements. Results show that no communication problems were present at a distance of 50 m while transmitting at the highest power mode.

With the results of experimental tests a comparative study of the power consumed by the wireless sensor nodes developed in this work and modules discussed in Section 2 was carried out. If for this analysis the relationship between wake-up and sleep state is considered, the system proposed in this work is better than the modules presented by other authors, as shown in Table 4. However, it is noteworthy that some of the WSNs analyzed in Section 2 only consider the state wake up and do not mention the sleep state. Another result obtained from the analysis is that, although there is enough data to do it, it cannot be determined exactly which system is best because there are differences between the modules analyzed like amount and type of elements, by example the solar cell used in Trio is RU6730 with a Pmax = 200 mW and Imp = 30 mA, which is different to the one used in this project.

## 7. Conclusions and Future Work

In this paper, we have presented a new solar powered model for the rapid prototyping of wireless sensor networks. The platform was developed as a tool for developers of WSN. One important feature of the Open-WiSe mote, among others, is the two 802.15.4 radios which allow developers to set up a rapid WSN application. Other important features of the WiSe mote include its robustness and low price. In addition, the schematics, gerber files, and PaRTiKle OS are available on the corresponding author's personal web page (http://www.siteldisolutions.com/index.php/es/descargas).

The solar powered model platform has been validated experimentally and the results show that by using sleep and wake-up strategies the lifetime of WSNs is increased. For future work we are developing a WSN gateway (802.15.4/802.11n), based on the open Hawk-board platform, to monitor and control physical variables through a Web-based platform.

## Figures and Tables

**Table 1.** Primary functions.

**Functions—Details**
void **sht75_read**(float *temperature, float *humidity)
**Returns:** The actual value of temperature and humidity.
void **adcInit0_6**(void)
Initiates an ADC conversion on P0.6 pin.
unsigned int **adcRead0_6**(void)
**Returns:** A 2 byte unsigned int value sampled by the ADC (10 bits).

**Table 2.** Current consumption at sleep and wake-up state for different duty-cycle rates (XBEE radio transceiver).

	**2%**	**10%**	**25%**	**50%**
*I_avg_* (mA)	7.52	12.45	21.89	37.25
*I_sleep_* (mA)	6.29	6.27	6.31	6.28
*I_awake_* (mA)	68.4	68.2	68.9	68.5
*I_node_* (mA)	7.5322	12.463	21.9575	37.39

**Table 3.** Current consumption at sleep and wake up state for different duty-cycle rates (CC2420 radio transceiver).

	**2%**	**10%**	**25%**	**50%**
*I_avg_* (mA)	3.2	6.39	12.65	23.6
*I_sleep_* (mA)	2.3	2.28	2.28	2.29
*I_awake_* (mA)	43.8	44.2	43.9	44.1
*I_node_* (mA)	3.13	6.472	12.685	23.195

**Table 4.** Energy consumed by WSN modules in wake-up and sleep states.

	**Trio**	**Heliomonte**	**Lee**	**Hande**	**Hwang**	**This paper**
*I_awake_/I_sleep_*	29.47 mA/NA	35.89 mA/NA	5 mW/NA	12 mA/9 mA	510 ma/NA	68.5 mA/6.28 mA
